# Advances and Future Challenges to Microbial Food Safety—Volume II

**DOI:** 10.3390/foods14132335

**Published:** 2025-07-01

**Authors:** Maria Lavilla

**Affiliations:** AZTI-BRTA, Food Research, 48160 Derio, Spain; mlavilla@azti.es; Tel.: +34-688-618-928

## 1. Introduction

Considering the increasing market demand for products with adequate nutritional, physicochemical and sensory characteristics [1], more efforts are being made to develop innovative approaches to manufacture food products with optimal characteristics while assuring their safety [2]. Recent advances in food production and processing technologies, as well as rapid detection methods, are key to limiting the emergence of foodborne disease [3,4].

However, microorganisms are highly adaptable and may survive and even grow in harsh food environments, and they can be easily transferred to food and people [5,6]. As a result, microorganisms remain a considerable cause of foodborne illnesses [7], leading to ongoing research and development regarding the application and optimization of emerging technologies, novel preservatives and natural antimicrobials to control foodborne pathogens from farm to table [8,9,10,11]. To disseminate research advances in these fields, reference publications are needed that provide alternative tools to manage microbial food safety throughout the entire food system. The second volume of this Special Issue, “Advances and Future Challenges to Microbial Food Safety”, aims to enhance knowledge in microbial monitoring and detail the latest advancements and novel strategies for pathogen control and inactivation (e.g., technologies, natural preservatives, and strategies to overcome challenges).

Among the 10 published papers included in this Special Issue, four main research topics are covered: (i) innovative treatments and technologies for microbial inactivation and pathogen control; (ii) antimicrobial properties of natural strategies, particularly essential oils; (iii) the growing capacities and virulence properties of microbial contaminants; and (iv) different parameters that affect the viability of relevant microorganisms with a potential impact on food safety.

## 2. Pathogen Inactivation

Three papers cover the first topic, discussing the effect of several innovative treatments and technologies for microbial inactivation and pathogen control (contributions 1–3). The first is an original study by Pinto et al. (contribution 1) that explores the impact of pH and high-pressure pasteurization on the germination and development of *Clostridium perfringens* spores under hyperbaric storage compared to refrigeration. It was performed in both culture media (BHI) at different pH levels and coconut water (pH 5.40) as a real case study. Unsurprisingly, acidic pH values (pH 4.5) in culture media inhibited the germination of spores, but inoculated coconut water with *C. perfringens* under atmospheric pressure and room temperature (AP/RT) conditions prompted rapid endospore germination and development. When subjected to HPP pasteurization, the initial spore load was slightly affected, as expected for this type of non-thermal treatment. After 15 days of storage at atmospheric pressure, at both RT and refrigerated temperatures, the microbial load increased. In contrast, when stored at hyperbaric pressure (75 MPa) at room temperature, the gradual decay of total microbial load was observed. The results of this study suggest that hyperbaric storage without temperature control can be used as a feasible alternative to refrigeration to avoid the development of *C. perfringens* spores and inactivate them without the application of high temperatures that are typically required to do so.

The second paper by Xu et al. (contribution 2) determined the antibacterial effect of combined ultrasound (US) and plasma-activated water (PAW) on the inactivation of *Aeromonas veronii*, a foodborne pathogen and spoilage microorganism commonly found in freshwater environments, to improve the preservation of crayfish. Initially, the study proved the efficacy of US-PAW in vitro in inactivating *A. veronii* and inhibiting its biofilm formation capacity. The authors additionally investigated the inhibitory mechanism of action of US-PAW against *A. veronii*. The results revealed changes in morphology and cell membrane damage, confirming that US enhances the harmful effects of PAW on outer cell membrane permeability, leading to more intracellular proteins and nucleic acids being leaked. Moreover, the combined treatment decreased antioxidant enzyme function and tricarboxylic acid cycle (TCA) efficiency, significantly compromising the antioxidant system and energy metabolism of the microbial cells and inducing bacterial death. When applied to crayfish, US-PAW was the most effective treatment against *A. veronii* and inhibited bacterial growth during storage, additionally preventing the formation of volatile basic nitrogen, an indicator of spoilage. As US-PAW treatment also preserved the flavor characteristics of crayfish during storage, the study concludes by outlining the potential of US-PAW as a novel preservation strategy.

The third publication by Romeo et al. (contribution 3) studies the microbial safety provided by sous vide (SV) cooking processes in chicken breast and egg recipes. Although this method offers new culinary possibilities, the use of temperatures below 70 °C also involves numerous risks, mainly microbiologically related, that must be assessed. As the authors exemplify, SV can serve as preservation technology, with average inactivation rates higher than 4 log against the studied vegetative microorganisms (*Salmonella* and *Campylobacter*). However, when considering specific microorganisms, the study suggests that treated products may still carry a potential microbiological risk as *Clostridum* spores in breast meat (1.70–2.30 log of inactivation) and *Salmonella* in eggs (which is still detectable via PCR after the treatments) may not be completely inactivated. These results highlight the need to avoid generalizations and instead analyze the microbial risks involved in specific conditions and recipes.

## 3. Antimicrobial Properties of Essential Oils

Two manuscripts focus on the antimicrobial properties of natural compounds, such as essential oils (contributions 4 and 5). Essential oils (EOs) are volatile plant extracts generally regarded as ecologically friendly and consumer-acceptable antimicrobial agents used for food preservation.

The first paper is an original study by Kyoui et al. (contribution 4)—a short communication that explores the antibacterial activity of hexanol vapor from three different commercial hexanol isomers against seven strains of food-related bacteria (*Escherichia coli*, *Pseudomonas aeruginosa*, *Salmonella enterica* Enteritidis, *Bacillus subtilis*, *Lactiplantibacillus plantarum*, *Listeria monocytogenes*, and *Staphylococcus aureus*). 1-hexanol exhibited antibacterial effects against Gram-negative bacteria at concentrations over 150 ppm, whereas Gram-positive bacteria were not affected by this compound. 2- and 3-hexanol did not demonstrate antimicrobial activity against any bacteria. When applied to vegetables, exposure to 300 ppm of 1-hexanol vapor decreased the total viable bacterial counts in approximately 3 logs in cabbage and carrot and inhibited bacterial growth in eggplants. Additionally, storage with exposure to 25 or 100 ppm of 1-hexanol vapor at lower concentrations (25 and 100 ppm) revealed the potential of this compound to act as an antibacterial agent; however, it also caused discoloration in cabbage. Therefore, further studies are required to improve its practical use.

The second paper by Zhang et al. (contribution 5) examines the inhibition mechanism of cinnamon essential oil (CEO) against *Aspergillus flavus* in walnut spoilage (rotting). The toxins produced by *Aspergillus* spp. pose significant health risks; as such, identifying the *Aspergillus* species present in walnuts and exploring the antifungal properties of natural compounds are crucial for developing guidelines to improve walnut storage and preservation. In the study, the authors isolated and examined more than 350 strains of the *Aspergillus* genus. Among them, *Aspergillus flavus* and *Aspergillus fumigatus* were the most frequent and dominant species (isolated in 100% and 93% of the samples, respectively). Then, the antibacterial properties and mechanism of action of CEO were systematically investigated on *Aspergillus flavus*. It was demonstrated that CEO inhibits mycelial growth in a dose-dependent manner. The in vitro minimum inhibitory concentration (MIC) and minimum fungicidal concentration (MFC) of CEO against *A. flavus* were determined to be 0.78 and 1.56 g/L, respectively. Cinnamon essential oil treatments can significantly disrupt the cell membrane integrity of *A. flavus*, causing increased relative conductivity, a higher release of cellular contents and a decrease in superoxide dismutase (SOD) activity (indicating increased intracellular lipid peroxidation). The authors conclude that CEO could be utilized as a natural bactericide in food preservation, although further research is needed to provide a broader view of its application.

## 4. Virulence Gene Expression and Growing Capacity

Two original articles and a review in this Special Issue address the growing capacities and virulence factors of microbial food contaminants (contributions 6–8). Yang et al. (contribution 6) explore the biofilm-formation capacity of different strains of *Listeria monocytogenes* and its effect on virulence and antimicrobial resistance. Although the study is limited to seven standard (collection) strains, it provides important data on the virulence factors of a highly relevant microorganism in the food industry. *Listeria monocytogenes* ATCC 19112 displayed the most potent biofilm-forming capacity and a self-agglutination rate of 32.69% at 5 h. However, no significant correlation was observed between cell surface hydrophobicity and biofilm formation when the seven strains were observed together. By contrast, the authors found that after biofilm formation, the adhesion and invasion of cells were enhanced and drug resistance increased. They established that the expression levels of genes associated with biofilm formation, including those involved in quorum sensing (QS), flagellar synthesis, and extracellular polymer production, were significantly upregulated after biofilm formation. Moreover, *L. monocytogenes*’ biofilm formation increased its resistance to 8 of the 14 antibiotics tested. These findings indicate that the formation of biofilm by *L. monocytogenes* increases its virulence, makes it more difficult to remove and improves its resistance to drugs, emphasizing the importance of effective biofilm control strategies in ensuring food safety.

Sousa et al.’s article (contribution 7) investigates the growth of three important food pathogens in co-culture, *Escherichia coli* O157:H7, *Salmonella* spp., and *Listeria monocytogenes*, to ascertain the most reliable enrichment method for their simultaneous detection. In culture media, selective *Salmonella Escherichia Listeria* broth (SEL) was the only tested medium that facilitated a slower but balanced growth of the three pathogens, reaching concentrations of about 10^8^ CFUs/mL for *E. coli* and *Salmonella* spp. and 10^5^–10^6^ CFUs/mL for *L. monocytogenes* at the end of a 24 h incubation enrichment process. Further assays were carried out in real conditions, with raw ground beef samples, to evaluate the performance of enrichment media in the presence of natural competing microbiota. Non-selective media limited the growth of *L. monocytogenes* due to the noticeable growth of competing microbiota and saturation effect of the medium. Again, the selective SEL medium was effective in preventing the overgrowth of fast-growing bacteria. This allowed for a sufficient concentration of the three pathogens to be reached, thus enabling the subsequent application of common detection procedures, such as PCR techniques. The authors claim that the most suitable enrichment broths should be always individually selected according to the properties (the limit of detection) of the detection technology.

The only review in this Special Issue, written by Rabiee et al. (contribution 8), addresses the implications for public health of microbial contamination and outbreaks associated with rockmelons (Cantaloupe). In recent years, these fruits have been linked to several serious foodborne illness outbreaks around the world: *L. monocytogenes* and *S. enterica* (different serovars) are the two main foodborne pathogens attributed to most disease outbreaks associated with rockmelon consumption since 2006. The microbiological contamination of rockmelons has caused six product recalls, resulting in significant economic losses for the food industry and subsequent business setbacks. The susceptibility of these fruits to microbial contamination is due to their close contact with soil during their final growth phase and their coarse outer skin, which provides an ideal environment for microorganisms to thrive. *Listeria*, *E. coli*, and *Salmonella* spp. easily attach to their rough and netted exterior, which, together with the high adaptability of these species, enables them to survive, persist and grow on the fruit surface. The authors provide general recommendations to reduce the contamination of pathogens using several best practice guides specific to melon production. They promote comprehensive food safety measures to consistently manage the food safety risks associated with the production and consumption of rockmelons to prevent future disease outbreaks and protect public health.

## 5. Microbial Viability

The final two papers in this Special Issue cover the fourth topic: different parameters that affect the viability of relevant microorganisms with a potential impact on food safety (contributions 9 and 10). The first by Jakopović et al. (contribution 9) is a predictive assessment of Ochratoxin A (OTA)’s effects on the oxidative stress parameters and fermentation ability of four wine yeasts: *Saccharomyces bayanus*, *Kluyveromyces marxianus*, *Hanseniaspora uvarum* and *Pichia guilliermondii*. The results indicate that OTA (4 µg/mL) initially affected the ethanol production of yeast, but after 24 h, no negative effect was observed in comparison to the control samples. However, OTA increased the two oxidative stress parameters studied. Glutathione (GSH) concentrations increased, even at low OTA concentrations, reflecting redox cellular protection mechanisms. Malondialdehyde (MDA) concentrations were also affected by higher OTA concentrations, indicating lipid peroxidation or cell damage. The authors attempted to use artificial neural networks (ANNs) to develop models that could predict the effects of different OTA concentrations on glucose utilization in ethanol production and GSH and MDA concentrations in yeast cultures. However, as there is still very little available information, further experiments are needed to improve the predictions made with ANNs.

Finally, the manuscript by Fedorowicz and Bartkowiak (contribution 10) studied the protective effect of six different butter samples on the viability rate of *Lacticaseibacillus rhamnosus* GG (LGG) immediately after spray-drying (SD) and during the storage of probiotic powders. In general, compared to the control sample, the addition of all tested butters had a protective effect on LGG during the SD process and during storage at both refrigerated (4 °C) and room temperatures (20 ± 1 °C). More specifically, the results showed that the highest viability of probiotic bacteria, after the spray-drying process and 4-week storage, could be indirectly influenced by a higher ratio of proteins and sugars to fat. Regarding the findings for reduced-fat butter (as well as salted and lactose-free butter), the higher viability of LGG seemed to be related (among other factors) to a lower content of palmitic acid (C16:0) and an increase in melting enthalpy. Altogether, the results verify the protective role of selected butters, potentially enabling their use in industrial processes to increase the durability of probiotic additives.

## 6. Conclusions

To conclude, the 10 papers published in this Special Issue serve to represent current research advances in tackling present and future challenges in microbial food safety. Most of the authors who have contributed promote the need for additional studies on this topic. Further research should explore the application of these technologies and strategies to other foodborne pathogens and a wider range of food matrices, optimizing their utility in food preservation and consumer protection against microbial risks in large-scale applications. This will additionally help us understand how microorganisms of treated products are inactivated under specific conditions.
